# Increased Great Saphenous Vein Diameter at the Level of Knee among Patients with Varicose Veins in a Tertiary Care Centre: A Descriptive Cross-sectional Study

**DOI:** 10.31729/jnma.7543

**Published:** 2022-08-31

**Authors:** Abhushan Siddhi Tuladhar, Sunil Pradhan, Amit Shrestha, Simant Shah, Rahul Shrestha

**Affiliations:** 1 Department of Radiology, Nepal Medical College and Teaching Hospital, Jorpati, Kathmandu, Nepal; 2 Nepal Medical College and Teaching Hospital, Jorpati, Kathmandu, Nepal

**Keywords:** *saphenous vein*, *ultrasonography*, *varicose veins*

## Abstract

**Introduction:**

Colour Doppler ultrasonography plays an important role in determining the morphological and hemodynamic information of the venous system. This study aimed to find out the prevalence of increased great saphenous vein diameter at the level of the knee among patients with varicose veins in a tertiary care centre.

**Methods:**

A descriptive cross-sectional study was carried out in the Department of Radiology at a tertiary care centre from 30 October 2021 to 31 March 2022 after taking ethical approval from the Institutional Review Committee (Reference number: 028-077/078). A convenience sampling technique was used for the study. The study group consisted of patients over 18 years, coming for ultrasonography examination of the lower limb with the clinical symptoms and signs of varicose veins. The great saphenous vein diameter was measured at the level of the medial femoral condyle of the knee using the software in the ultrasonography unit. B mode, colour Doppler and spectral analysis were done. A cut-off value of 5 mm for the diameter of the great saphenous vein was taken to indicate the presence or absence of varicosity and saphenofemoral reflux. Point estimate and 90% Confidence Interval were calculated.

**Results:**

Among 72 patients with varicose veins, the diameter of the great saphenous vein was increased in 59 (81.94%) (74.50-89.38, 90% Confidence Interval) patients.

**Conclusions:**

The mean diameter of the great saphenous vein in our study was similar when compared to other studies conducted in similar settings.

## INTRODUCTION

Varicose veins are tortuous, dilated, and painful veins that have filled with an abnormal pooling of blood.^1^ The most common manifestation of chronic venous insufficiency is the varicose vein, normally due to abnormal dispensability of connection without prior thrombosis.^2^ Reflux in the venous system plays an important role in the progression of chronic venous insufficiency.^3^ Among the non-invasive procedures, colour Doppler Ultrasonography (USG) is used for precise localization of valvular incompetence and venous thrombosis.^4,5^

Studies have shown that the Great Saphenous Vein (GSV) with a diameter of less than 5 mm tends to have a lower incidence and a diameter of more than 5 mm tends to have a higher incidence of venous insufficiency.^6,7^ However, studies on this condition are scarce in Nepal.

This study aimed to find the prevalence of increased great saphenous vein diameter at the level of the knee among patients with varicose veins in a tertiary care centre.

## METHODS

A descriptive cross-sectional study was carried out in the Department of Radiology of Nepal Medical College and Teaching Hospital from 30 October 2021 to 31 March 2022. The study population consisted of all the patients coming for USG examination of the lower limb with the clinical symptoms and signs of varicose veins. The study was started after taking ethical approval from the Institutional Review Committee (Reference number: 028-077/078), and also after taking informed consent.

The study group comprised patients coming for ultrasonography examination of the lower limb with the clinical symptoms and signs of varicose vein and were screened using inclusion and exclusion criteria. Patients over 18 years of age and patients clinically suspected of presence of GSV reflux and varices were included in the study. The patients excluded from the study were patients less than 18 years, patients with deep vein thrombosis, cases of varicose veins with its complications (thrombosis, phlebitis, cellulitis, venous ulcers), and cases of varicose veins with other vascular pathology of the limbs occurring at the same time like a-v malformation, hemangioma, previously treated cases for varicose veins like surgery, LASER therapy. A convenience sampling method was used. The sample size was calculated by using the formula


$$\begin{array}{ll} \text{n} & ={\text{Z}}^{2}\times \frac{\text{p}\times \text{q}}{{\text{e}}^{\text{2}}}\\ & =1.64^{2}\times \frac{0.50\times 0.50}{0.10^{2}}\\ & =68 \end{array}$$

Where,

n= minimum required sample size
Z= 1.64 at 90% Confidence Interval (CI)
p= prevalence taken as 50% for maximum sample size
q= 1-p
e= margin of error, 10%

The minimum required sample size was 68. However, a sample size of 72 was taken. The patients were scanned using a Toshiba Nemio 17 ultrasound unit with a 7-megahertz convex transducer. To avoid observer bias, only the faculty members involved in this study performed the sonographic scan. GSV diameter was measured at the medial femoral condyle at the level of the knee using the software in the USG unit. B mode, colour Doppler and spectral analysis were done. The studies were done in the standing position with weight-bearing by the contralateral limb. Both limbs were evaluated where there was a bilateral clinical indication. A cut-off value of 5 mm was taken for the diameter of the great saphenous vein to correlate with the presence or absence of varicosity and saphenofemoral reflux.^6^

The inner anechoic diameter of the GSV was measured by holding the probe transversely without pressure. Colour Doppler sonography was used to evaluate the flow direction with distal compression and release, and reflux was quantified based on the valve closure time as seen on Doppler spectral tracings. Reflux was defined if the valve closure time was more than 0.5 seconds, as recently recommended by a study.^3^

Data were entered and analysed in IBM SPSS Statistics 21.0. Point estimate and 90% CI were calculated.

## RESULTS

**Figure 1:**
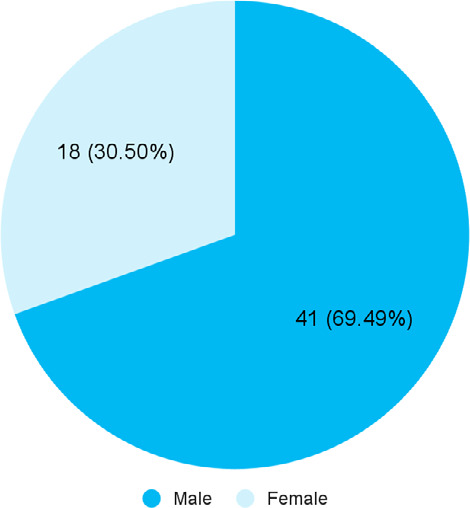
Sex-wise distribution of the patients with increased GSV diameter (n= 59).

Among 72 patients with varicose veins, the diameter of the great saphenous vein was increased in 59 (81.94%) patients (74.50-89.38, 90% CI). The mean age of the patients was 47.76±3.43 years with male predominance (Figure 1).

The GSV diameter was increased most commonly in the 41-60 years of age group in 25 (42.37%) (Table 1).

**Table 1:** Distribution of age and gender in patients with increased GSV diameter (n= 59).

Age groups	Male n (%)	Female n (%)	Total n (%)
18-40 years	16 (27.12)	5 (8.48)	21 (35.59)
41-60 years	14 (23.73)	11 (18.64)	25 (42.37)
61-80 years	6 (10.17)	1 (1.69)	7 (11.86)
> 80 years	5 (8.48)	1 (1.69)	6 (10.16)

The prevalence of varicose veins was found more in patients with occupations requiring physical exertion and hardship, long-standing and strenuous jobs with the highest number of patients (Table 2).

**Table 2:** Increased GSV diameter with different occupations (n= 59).

Occupation	Varicosity n (%)
Farmer/fieldwork/agricultural work	10 (16.95)
Teacher	2 (3.39)
Army/police/security guard	12 (20.34)
Driver	5 (8.48)
Labourer/mason/carpenter/plumber/helper	15 (25.42)
Housewife	11 (18.64)
Athletes/sportsmen	1 (1.69)
Intellectual/table work	3 (5.09)

Among 59 patients having GSV diameter of 5 mm or more, 56 (94.91%) had incompetent Sapheno-femoral Junction (SFJ) (Table 3).

**Table 3:** Increased GSV diameter and SFJ incompetence (n= 59).

Colour Doppler study findings	GSV diameter >5 mm
Sapheno-femoral junction competent	3 (5.08)
Sapheno-femoral junction incompetent	56 (94.92)

Color Doppler study showed that among 59 patients with raised GSV diameter, the saphenofemoral junction was incompetent in 56 (94.91%), the saphenopopliteal junction was incompetent in 51 (86.44%) and the perforators above and below the knee were incompetent in 5 (8.47%) (Table 4).

**Table 4:** Colour Doppler study findings among patients with GSV more than 5 mm (n= 59).

Color Doppler study findings	n (%)
Competent saphenofemoral junction	3 (5.08)
Incompetent saphenofemoral junction	56 (94.92)
Competent sapheno-popliteal junction	8 (13.56)
Incompetent sapheno-popliteal junction	51 (86.44)
Incompetent perforators above the knee	7 (11.86)
Incompetent perforators below the knee	37 (62.71)
Incompetent perforators above and below the knee	5 (8.48)
No incompetent perforators	10 (16.95)

## DISCUSSION

In this study, the prevalence of increase in the diameter of the great saphenous vein was 81.94% patients. In normal veins, the venous valves keep blood moving forward toward the heart. In varicose veins, the valve malfunctions, causing blood to pool in the vein, which ultimately causes the vein to widen. Some of the most common causes are congenitally defective valves, pregnancy, and thrombophlebitis. Standing for an extended period of time, several months or years, with increased intra-abdominal pressure is likely to lead to varicose veins, or worsen the condition. This process usually takes place in the veins of the legs, although it can occur in different parts of the body.^2^

Chronic venous diseases of the legs are common in the Western general population.^8^ In this study, the mean age of the patients was 47.76±3.43 years with male predominance estimates of the prevalence of varicose veins range widely from 2-56% in men and 1-60% in women.^9^ Varicose veins are common, affecting primarily women.^10^ These variations reflect differences in study population variability, including age, race, gender, methods of measurement, and disease definition.^11-13^

Varicose veins are veins that have become dilated and tortuous.^14^ The veins have valves that prevent the backflow of the blood. The leg muscles pump the veins to return blood to the heart, against the force of gravity.^15^ In the varicose vein, the valve leaflets don't stick together properly and the valves do not work, eventually leading to a backflow of blood, causing the vein to enlarge. Varicose veins are most common in the superficial veins of the legs, which are exposed to high pressure when standing.^16^ In addition to cosmetic problems, varicose veins are often painful, especially when standing or walking.^14^

Excess weight, heavy lifting, and pregnancy also increase the likelihood of developing varicose veins as they all put increased pressure on the body.^13^ Aging, menopause, genetic weaknesses in the walls of the veins, excessive pressure within the veins due to a low fiber diet causes an increase in straining to pass stool, and damage to the veins or their valves resulting from inflammation also increases the risk of developing varicose veins.^13^

The prevalence of varicose veins among male and female patients in this study was 69.49% and 30.50% respectively which was not comparable to the Framingham Study in 1988.^17^ This may be because of obesity, lower level of physical activity, sedentary activities, and higher age at menopause all contributed to the development of varicose veins among women in their study, which does not hold in the Nepalese context.

In this study, a higher number of varicose veins was seen in occupations demanding heavy physical exertion, load-lifting and hardship, prolonged standing, and walking. Another study also described the same factors as the main causes of varicosity.^13^ In this study, 81.94% of patients had a GSV diameter of >5 mm and only 18.06% had <5 mm. The mean diameter of GSV was 5.6±0.13 mm. Among all patients with GSV >5 mm, 94.91% had incompetent SFJ and 5.09% had competent SFJ. This is similar to other studies.^6,7^ A study concluded that a GSV diameter of less than 5 mm has a tendency to have a lower incidence of venous incompetence and a GSV diameter of greater than 5 mm has a tendency to have a higher incidence of venous incopetence.^6^

A study concluded that GSV diameter measured by B mode imaging proved to be a relatively accurate measure of hemodynamic impairment and clinical severity in a model of SFJ and GSV incompetence predicting not only the absence of abnormal reflux but also the presence of critical venous incompetence assisting in clinical decision making before considering greater saphenectomy.^7^

Other colour Doppler findings of patients with varicose veins in this study were also similar to the findings in a study that concluded that the extent of great saphenous veins insufficiency correlated with an increase in the number and the diameter of perforators.^18^ In this study, among all patients with an increased GSV diameter of more than 5 mm, besides having more incidence of the incompetent saphenofemoral junction, 100% had varicose veins, 86.44% had incompetent saphenopopliteal junction and 83.01% had incompetent perforators either above the knee or below the knee or both above and below the knee.

The study had some limitations. There could be interobserver variability between the measurements of the great saphenous vein diameter. As this was a single-centric study, the findings of this study cannot be generalised to the entire population of Nepal. Also, causality or association between the variables could not be established in this study design. Lastly, the sample size of our study was also small.

## CONCLUSIONS

The mean diameter of the great saphenous vein at the level of the knee among patients with varicose veins in our study was similar when compared to other studies conducted in similar settings. Colour Doppler ultrasonography could be a reliable tool for the precise localization of valvular incompetence and venous thrombosis and could be beneficial in early diagnosis and management.
